# Bioinformatic analysis of a novel *Echinococcus granulosus* nuclear receptor with two DNA binding domains

**DOI:** 10.1371/journal.pone.0224703

**Published:** 2019-11-11

**Authors:** Gabriela Alvite, Ximena Riera, Saira Cancela, Margot Paulino, Adriana Esteves

**Affiliations:** 1 Biochemistry Section, Faculty of Sciences, Universidad de la República, Montevideo, Uruguay; 2 Center of Bioinformatics, Departamento de Experimentación y Teoría de la Materia, Faculty of Chemistry, Universidad de la República, Montevideo, Uruguay; Université de Genève, SWITZERLAND

## Abstract

Nuclear receptors are ligand-activated transcription factors capable of regulating the expression of complex gene networks. The family includes seven subfamilies of proteins with a wide phylogenetic distribution. A novel subfamily with two DNA-binding domains (2DBDs) has been reported in *Schistosoma mansoni* (*Platyhelminth*, *Trematoda*). This work describes the cDNA cloning and bioinformatics analysis of Eg2DBDα, a 2DBD nuclear receptor isoform from the parasite *Echinococcus granulosus* (*Platyhelminth*, *Cestoda*). The *Eg2DBD*α gene coding domain structure was analysed. Although two additional 2DBD nuclear receptors are reported in the parasite database GeneDB, they are unlikely to be expressed in the larval stage. Phylogenetic relationships between these atypical proteins from different cestodes are also analysed including *S*. *mansoni* 2DBD nuclear receptors. The presence of two DNA binding domains confers particular interest to these nuclear receptors, not only concerning their function but to the development of new antihelminthic drugs.

## Introduction

The field of nuclear receptors (NRs) has undergone an astounding evolution since the biochemical identification of the first nuclear receptor in the early of the 1960's [1]. Nevertheless, important gaps in our knowledge remain to be addressed, concerning NRs action, ligands, co-activators and co-repressor proteins and other components of the transcriptional machinery.

NRs belong to a large protein superfamily of ligand-activated transcription factors that bind specifically to DNA sequences. This family includes receptors for steroid hormones, thyroid hormone, vitamin D, retinoids, fatty acids and prostaglandins, which bind hydrophobic ligands and regulate a variety of mammals' genes [2–3]. A large number of them have no defined ligand and are hence described as orphan receptors [2, 4–5]. Receptors of this family have been reported in insects, worms and amphibians, in addition to mammalian species [6]. According to phylogenetic studies, NRs emerged long before the divergence of vertebrates and invertebrates, during the earliest metazoan evolution [7].

Based on the sequence alignment of conserved domains and phylogenetic tree construction, the Nuclear Receptor Nomenclature Committee has classified the nuclear receptor gene family into seven subfamilies (NR0-NR6) [8]. Recently, a new subfamily named 2DBD-NR with members that contain two DNA binding domains (2DBDs) was reported [9]. Members of this new subfamily were identified only in invertebrates (Mollusca, Arthropoda and Platyhelminthes). The authors also suggested a close evolutionary relationship with receptors of the NR subfamily I receptors.

NRs have modular organisation, with various degrees of conservation among their respective domains. Typical NRs contain an N-terminal domain (region A/B), a DNA binding domain (DBD, region C), a hinge domain (region D), a ligand binding domain (LBD, region E) and a C-terminal region (region F) [10–12]. The DBD, the most conserved region, contains a P-box, a short motif responsible for direct DNA interaction and DNA-binding specificity, and a D-box implicated in dimerisation pattern. The C-terminal region of the DBD (CTE) has two other conserved motifs, the T-box and the A-box, involved in DNA recognition and dimerisation respectively [13]. CTE sequences are not conserved among NRs, and adopt different structural motifs. However, these divergent structures share a common function to extend the protein-DNA interface beyond that of base-specific contacts in the minor groove thus stabilising DNA binding [14].This region could have a role in selective binding to targets promoters. A region termed GRIP-box with similar localisation and functions of the T-box was also reported [15]. The ligand-binding domain is less conserved but contains two highly conserved regions, the LBD signature Tτ and the AF2 motif related to activation. The LBD also contains a strong dimerisation interface and a ligand-binding pocket [16]. The F region is not present in all receptors and its function is poorly known [11].

*Echinococcus* species are the smallest tapeworms of medical significance and, similar to tapeworms, require two mammalian hosts, including man, for completion of their life cycle. *E*. *granulosus* is universally distributed, representing in many countries a major public health problem. Its life cycle begins when eggs containing the embryo (onchosphere), are shed with the faeces of the definitive host (canids). Once a suitable intermediate host ingests the eggs, they hatch and the onchosphere is released, entering the circulation. At the final site of infection, the larval stage may progress to form a hydatid cyst. The cyst produces thousands of protoscoleces, which can progress to the adult form when the cyst is ingested by the definitive host [17].

The first Platyhelminth NRs were identified in *Schistosoma mansoni* [18–22]. The advent of genome projects of cestodes has opened doors for the studies in these organisms. Interested on *E*. *granulosus* fatty acid binding protein nuclear partners we initiated the search of a PPARα-like nuclear receptor in GeneDB database. The best hit was EgrG 000379600.1. which belongs to the new 2DBD-NR subfamily of nuclear receptors.

In this study, we report the cloning and bioinformatics analysis of a new isoform of *E*. *granulosus* nuclear receptor gene from protoscolex larval stage. In addition, domains conserved signals as well as phylogenetic relationships between 2DBD-NRs members of cestodes were analysed and compared with known *S*. *mansoni* members of this family of proteins. A three dimensional model was performed as a first approach to analyse how this atypical NR interact with DNA. This is the first report of cestodes' 2DBD-NRs analysis, which contributes to the knowledge of these atypical nuclear receptors.

## Materials and methods

### Organisms

Fertile hydatid cysts from *E*. *granulosus* G1 strain were collected during the routine work of local abattoirs in Montevideo (Uruguay) from the lungs of naturally infected cattle. The hydatid cyst fluid with protoscoleces was aseptically aspirated according to Esteves and collaborators [23]. Once settled by gravity, the protoscoleces were extensively washed in phosphate-buffered saline (PBS) to remove dead protoscoleces debris and were observed under a light microscope for viability (flame cell and vital dye exclusion). Finally, the material was kept frozen at -80°C in TRizol^TM^ Reagent (ThermoFisher Scientific) until use.

### Identification of putative nuclear receptors of *E*. *granulosus*

A search of sequences of nuclear receptors was performed in the GeneDB database (www.genedb.org) using *Mus musculus* peroxisome proliferator-activated receptor PPARα amino acid sequence as the template. Three sequences with high score were identified. The following primers were designed to amplify each coding region from the conserved DBD to the stop codon: Fw37: 5'-TGTCGGGTCTGCGGTGGACG-3'; Rev37: 5’-CGAGGACGTCAACCATCTACG-3'; Fw24: 5'-TGCCGCGTTTGTGGCGCGCA-3'; Rev24: 5'-TCACTCGCTCGAATTAACCAA-3'; Fw45: 5'-ACGACAACTTTGCACATGTT-3'; Rev45: 5'-AAGTCATTCCTGTTGGAATCGTTCC-3'.

### RNA isolation and cDNA synthesis

Total RNA was isolated from protoscoleces using TRizol^TM^ Reagent according to the manufacturer's instructions. Retrotranscription was performed using RT Superscript III (Invitrogen), with 200 ng of random primers and 4.6 μg of total RNA. Total RNA was DNA digested with RQ1 RNase-Free DNase (Promega), following the manufacturer's instructions.

### Polymerase chain reaction

Polymerase chain reaction (PCR) was performed using a KAPA HiFi HotStart kit (KAPA BIOSYSTEMS) with a final volume of 25 μl, 10 pmol of forward and reverse primers (Fw37-Rev37, Fw24-Rev24, Fw45-Rev45), 0,8 mM dNTPs, 1x KAPA buffer, 1,5 mM MgCl2, 1 μg *E*. *granulosus* protoscoleces cDNA and 0,5 U of the enzyme. Reaction conditions were 3 min at 95°C followed by 35 cycles of 20 sec at 98°C, 30 sec at 66°C, 120 sec at 72°C, with a final extension of 4 min at 72°C. Negative controls were included. The absence of contaminant genomic DNA was controlled through the amplification of a known gene (*egfabp1)* having an intron [23]. The unique PCR product obtained was fractionated by 1% agarose gel electrophoresis, the band was excised from the gel and purified with a Universal DNA purification kit (Tiagen). This product was subjected to a second amplification using RANGER DNA polymerase (RANGER Mix, BIOLINE) and the same set of primers, following the manufacturer's instructions. The same cycling conditions were employed, except for primers annealing temperature (63°C), the final extension (72°C x 5 min) and the number of cycles (30). After the re-amplification step the DNA band was purified from the agarose gel as mentioned above and sequenced at Macrogen Service (Korea). This PCR product matched with EgrG 000379600.1 sequence. In addition, the following primers were designed to amplify the lacking 5´ EgrG 000379600.1 coding region: 5´-ATGGCACAAACACCTGCAGCCACAG-3´ (Eg2DBD-1F) and 5´-GATGAGTTTGTCCGCCAGAG-3´ (37_MSLrev).

### Cloning

Both PCR products from EgrG 000379600.1 were cloned to the pGM-T vector (pGM-T ligation Kit, TianGen) according to the manufacturer's instructions. After that, three clones of each cloning reaction were verified by colony PCR and the purified plasmids were sequenced at Macrogen Service (Korea), using universal primers (T7 and SP6 promoter), and specific primers (HKWRev: 5'-TTCTAGACGGCTCCACTTATG-3'; VPQRFw: 5'-GTACCACAGATGCCTATCAC-3') and those mentioned to amplify the 5´ region.

### Bioinformatics studies

Nucleotide sequence was analysed using Chromas v.1.43 software (http://www.technelysium.com.au/chromas.html) and manually corrected when necessary. Sequence alignment (DNA or cDNA) were performed using CLUSTAL Ω algorithm under default conditions [24]. Gene organisation was determined by cDNA alignment with the genomic DNA sequence obtained from the GeneDB database.

2DBD-NR protein sequences from *Echinococcus multilocularis*, *Mesocestoides corti*, *Hymenolepis microstoma*, *Schistosoma mansoni* and *Taenia solium*, were obtained from GeneDB WormBase Parasite (https://parasite.wormbase.org) using the CLUSTALΩ algorithm under default conditions (https://www.ebi.ac.uk/Tools/msa/clustalo/).

Bioinformatics predictions were performed to identify phosphorylation, SUMOylation (http://gps.biocuckoo.cn/), and cellular localisation motifs (Predict Protein, https://ppopen.rostlab.org). PROSITE (http://prosite.expasy.org), SMART (http://smart.embl-heidelberg.de) and Pfam (http://pfam.xfam.org) domain databases were employed to locate EgNRs domains.

### DNA binding domains structure

The amino acid sequence of Eg2DBDα was employed to search structurally homologous sequences in the protein data bank (PDB) using the sequences annotated by structure (SAS) server available at EBI [25]. The amino acid similarity between the target (query) and the best templates was observed to be less than 40%.

Two templates were selected to model the DBDs: 2FFO to model DBD I and 2NLL to model DBD II. Ab Initio models were obtained by The Iterative Threading Assembly Refinement (I-TASSER) server [26] with the following parameters (DBD I: TM-score = 0.78, C-score = 0.51, RMSD = 2.8Å; DBD II: TM-score = 0.77, C-score = 0.44, RMSD = 2.9Å) and refined by means of an energy minimization in MOE ChemComp suite package (MOE—Molecular Operating Environment, Chemical Computing Group, Montreal, 2014), ETH-Amber12 force field, gradient conjugate algorithm with a convergence gradient of 0.01 kcal/mol. Both structures were superposed with their pdb template hits, 2FF0 and 2NLL respectively. The new coordinates of the DBDs were saved, together with the double strand DNA structure from 2NLL in order to show a possible binding/mechanism of interaction between the two macromolecules. PROCHECK was then used to analyse the structural and stereo-chemical properties of the domains by recognizing overall and residue-by-residue geometry. A Psi/Phi Ramachandran plot was used to assess the quality of the model. The reliability of the model was assessed via ERRAT, which examines the statistics of non-bonded interfaces between diverse atom types.

### Phylogenetic studies

A rooted phylogenetic tree using programs from the MEGA (X) package and amino acid data sets for both DBDs of cestodes and *S*. *mansoni* 2DBDα, 2DBDβ, 2DBDγ was constructed [27]. Sequence alignment was performed using the CLUSTAL ^ algorithm software under default conditions. The topology and the branch lengths of the phylogenetic tree were estimated using the Maximum Likelihood method under Jones-Taylor-Thornton (JTT) substitution model, with a discrete Gamma distribution to model evolutionary rate differences among sites (eight categories (+G, parameter = 1.4068)) [28]. The significance of branching points was assessed by bootstrapping with 100 replicates. As an external group sponge NR sequence was included.

## Results

### Primary structure analysis

**Fig 1:**
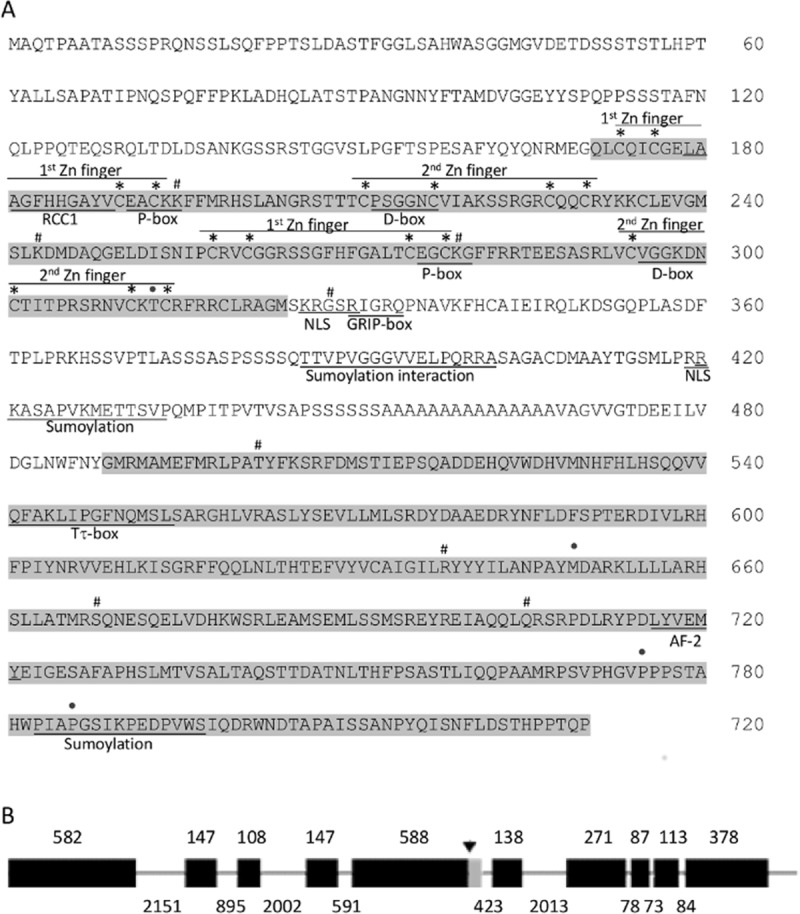
Eg2DBDα sequence analysis. A) Amino acid sequence of Eg2DBDα. DNA binding domain and ligand binding domain are shadowed; consensus motifs are underlined. *, # and • indicate Cys of fingers, splicing sites and substitutions, respectively. B) Schematic representation of intron-exon organization. The numbers indicate pair of bases. Black boxes: exons; grey box: deleted region from annotated sequence; line: introns.

Three *E*. *granulosus* 2DBD nuclear receptor sequences were extracted from the GeneDB database using *Mus musculus* PPARα as a template, with the following accession numbers: EgrG 000379600.1 (Eg37), EgrG 00240200 (Eg24) and EgrG 000458200 (Eg45). Although three specific primer sets were assayed, only one positive PCR amplification product from protoscoleces cDNA was obtained. The cloned 5´ coding region overlaps 843 bp with the previously mentioned fragment. The identity of both fragments with EgrG 000379600.1 coding sequence was verified by alignment showing some differences explained below. The cDNA sequence reported here was deposited in GenBank under the accession number MH092994.2 and the coding protein as AZM65758.2 (Eg2DBDα). The full length sequence of 2493 bp encodes 830 residues. Comparison with the database sequence (Eg37) revealed some mismatches that lead to amino acid substitutions: A^313^ to T, T^649^ to M; S^775^ to P and S^786^ to P. A deletion of 22 residues was also observed. The translated sequence of the cloned nuclear receptor is depicted in Fig 1A. The genomic annotated sequence was compared with the translated sequence in order to identify splicing sites (Fig 1B). Gene structure contains nine introns and ten exons expanding over 10868 bp that encodes an 830 amino-acid protein.

**Fig 2:**
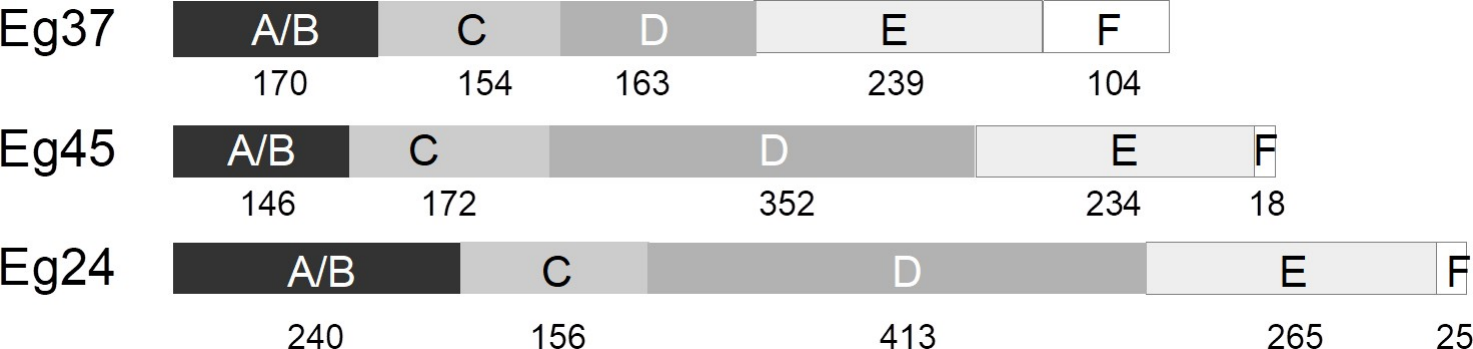
Functional domains of 2DBD nuclear receptors of *Echinococcus granulosus*. Schematic representation of Eg2DBDs domains according to PROSITE database. A/C: Nt domain; C: DNA binding domain; D: hinge region; E: ligand binding domain; F: C-terminal region. The size of each domain in amino acids is indicated. Sequences from Eg24 (EgrG 00240200) and Eg45 (EgrG 000458200) were retrieved from GeneDB database.

Several approaches were performed in order to define the topological organization of the identified 2DBD nuclear receptors: SMART, PROSITE and alignment with known structures. All of them matched the DBD location but differed in the LBD identification, probably because it is a less conserved region and each tool employs different signatures for the identification of this domain. Fig 2 shows the domain organisation of the three *E*. *granulosus* 2DBD nuclear receptors according to the PROSIT.E tool.

UniProt analysis showed that the protein Eg2DBDα has two DBDs. The first DBD is predicted to be composed of two Zinc fingers with the conserved sequences Cys X_2_Cys X_13_ Cys X_2_ Cys and Cys X_5_ Cys X_9_ Cys X_2_ Cys respectively, and the consensus P-box “EACKK” identified in *S*. *mansoni* [29]. The second domain also contains two Zinc fingers with almost the same pattern of the first DBD (Cys X_2_Cys X_13_ Cys X_2_ Cys and Cys X_6_ Cys X_9_ Cys X_2_ Cys). Consensus P-box and D-box motifs were also identified (Fig 1).The first core DBD of 68 residues has 73% similarity and 41% identity with the second domain of 67 residues (typical core NR-DBDs are 66–70 residues). C-terminal extension (CTE region) sequences are not conserved among nuclear receptors. The lack of reported consensus sequences makes its precise identification difficult. The length of the hinge region is not easy to distinguish since no consensus regions are defined. They are usually short regions (40 amino acids approximately in most NRs) that allow flexibility between the two domains. However, the length of the hinge in Eg2DBDα can be estimated to be 163 amino acids and a similar estimation (156 residues) was obtained through PROSITE database.

Several papers have emphasized that in addition to the C domain, two regions of the LBD of vertebrate nuclear receptors are conserved, T signature and AF-2. The consensus sequence of the Tτ signature is φAKXhPXFXXLXXXDQXXLL where φ is an aromatic residue and h a hydrophobic one [16]. *E*. *granulosus* 2DBD cloned receptor Tτ region does not fit completely but is under the 20%-45% identity reported for vertebrate NR sequences [30] (Fig 1A).

Sequence alignment with solved LBD structures is particularly difficult since this region has low conservation, particularly between cestodes and vertebrates. However a careful visual inspection allowed us to detect a residue specific of class II NRs, and the absence of the specific residue of class I, reported by Brelivet et al. in 2004 [31]. The mentioned residue is a glutamic acid located at position 668 of Eg2DBDα. Anyway, as long as we have not solved the 3D structure of the molecule, we cannot say if belongs to this class of receptors. A typical AF-2 sequence was identified at residues ^716^-LYVEMY-^721^. The AF-2 consensus sequence is ZZXEZZ where Z is any hydrophobic amino acid [32].This motif is found in *S*. *mansoni* Sm2DBDα and Sm2DBDβ sequences [29].

The prediction algorithm from Predict Protein, localises the protein inside the nucleus. Considering this prediction and knowing that nuclear receptors should be shuttled from the cytoplasm to the nucleus we searched for a putative nuclear localisation signal (NLS). Eg2DBD under study does not contain the "classical" NLS from the SV40 large T antigen [33–34]. Since more complex nuclear localisation signal sequences than "classical" NLS have been reported, we propose two putative NLSs composed of residues ^325^KRX2R^329^ and ^419^RRK^421^ located at the putative hinge binding domain [35–37].

The Nt region and DBDs are also the target of post-translational modifications, particularly phosphorylation and SUMOylation. The search of the predicted sites for these modifications showed so many putative phosphorylation sites that is not possible to propose any of them. Consensus SUMOylation motifs could not be found in these regions. A RCC1 consensus motif (LAAGfHHGAYV) was identified in the first zinc finger of DBD I (residues 179–189).

For hinge region it is interesting to mention the presence of a long tract of Ser-Ala. An alanine tandem could confer a considerable flexibility to the region. Our analysis also allowed us to identify a motif similar to the GRIP-box of orphan receptors. The consensus sequence is RXGRZP where Z represents a hydrophobic amino acid while Eg2DBDα motif is ^330^RIGRQP^335^ where the hydrophobic consensus amino-acid is substituted by the polar residue Q [15] (Fig 1A). As previously mentioned, this motif could be considered the T-box of the CTE region.

Bioinformatics analysis also predicted, two SUMOylation sites at positions 427 and 791 (^420^RKASAPV**K**METTSVP^434^, ^783^PIAPSGSI**K**PEDPVWS^798^), and a SUMOylation interaction region at positions 394–398 (^387^TVPVGGG**VVELP**QRRASAG^405^) (Fig 1A).

### DNA binding domains structure

**Fig 3:**
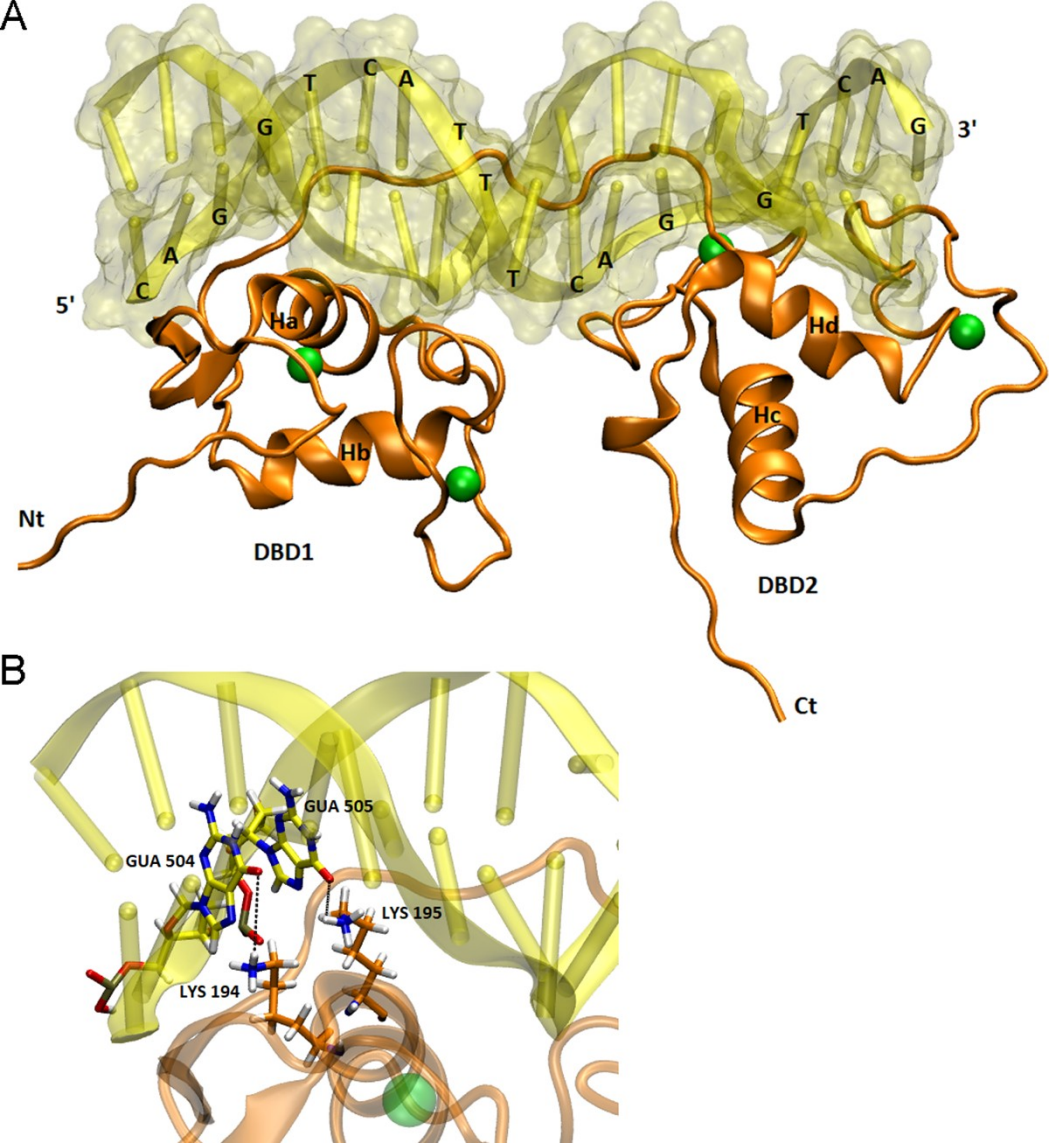
Molecular model of Eg2DBDα DNA binding domains. A) DBDs-DNA complex. B) Amplified view of DBD I-DNA interaction. In orange ribbons are depicted the secondary structures of DBD domains. DNA is shown as a molecular surface containing direct repeats of the canonical AGGTCA half-site separated by four base pairs. Zn atoms are shown as green balls. Hydrogen bonds between base pairs and residues from DBDs are indicated by dotted black lines.

The structure of Eg2DBDα DNA binding domains bounded to DNA is shown in Fig 3. Quality assessment via ERRAT and PROVE indicated that the model statistics were appropriate, consequently validating the built model. Thus, model validation suggested that the model adequately represented the native protein. Taking into consideration that there is no biological evidence of how this new class of nuclear receptor interact with the DNA in the cell and none crystallographic structure was reported, here we present a reliable model predicting one possible way of interaction between the Eg2DBDα DNA binding domains and a the response element from the crystal structure of PDB ID. 2NLL (Fig 3).

The face of DBDs that interacts with DNA contains a positive electrostatic charge potential that complements with the negative electrostatic potential of the DNA. The alpha-helix Ha of the DBD I engage the major groove side of the DNA, making base-specific interactions with the response element through de P-box motif. In particular, Lys194 and Lys195 act as hydrogen donor to establish hydrogen bonds with DNA bases at positions G504 and G505. In addition, the DBD II interacts with DNA but not through the P-box contributing to the stabilization of the complex.

### Identification of 2DBD-NRs in other cestodes

**Fig 4:**
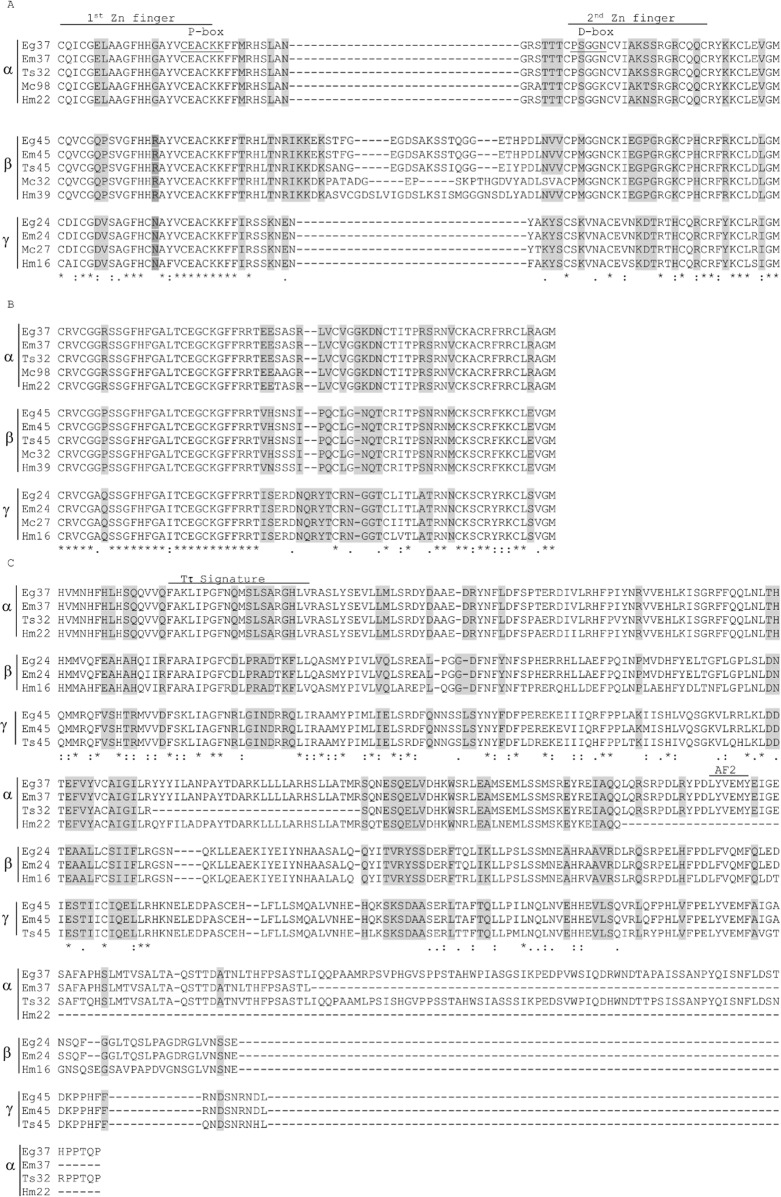
Sequence alignment of cestodes 2DBD nuclear receptor domains. A) DBD I; B) DBD II; C) LBD. Abbreviations and accession numbers of the aligned sequences are: Eg37: EgrG 000379600; Em37: EmuJ_000379600.1; Ts32: TSA 0003204923; Hm22: HmN_002208800.1; Mc98: MCOS_0000009801; Egr45: EgrG 000458200; Em45:EmuJ_000458200.1: Ts45:TSAS00045G06070; Hm39: HmN_000395600.1; Mc32: MCOS_0000321501; Eg24: EgrG 00240200; Em24: EmuJ_000240200.1; Hm16: HmN_000166400.1; Mc27: MCOS_0000027401. The shaded residues are specific of each cluster. Clustal Ω identity and similarity nomenclature is showed. For space reasons we do not include the complete sequence of the LBDs. Groups are indicated as α, β, and γ.

A BLASTP search with Eg37 as the template through the GeneDB and WormBase Parasite databases led us to identify 2DBD nuclear receptors from other cestodes: *Mesocestoides corti* (MCOS_0000009801, MCOS_0000321501, MCOS_0000027401) *Hymenolepis microstoma* (HmN_002208800.1, HmN_000395600.1, HmN_000166400.1), *Taenia saginata* (TSA 0003204923; TSAS00045G06070), and *Echinococcus multilocularis* (EmuJ_000379600.1, EmuJ_000240200.1 and EmuJ_000458200.1). Alignment of DBD and LBD regions of these protein sequences showed specific residues that suggests that these receptors can be clustered in three groups (Fig 4). *M*. *corti* sequences were not included in the LBD alignment since the presence of long tracks of mismatches suggesting that the reference database has not been curated.

P-boxes and finger patterns (C-X_2_-C-X_13_-C-X_2_-C and C-X_5_-C-X_9_-C-X_2_-C; C-X_2_-C-X_13_-C-X_2_-C and C-X_6_-C-X_9_-C-X_2_-C) were conserved in all cestodes receptor sequences analysed. However, the first DBD domain has lower sequence similarity (22%) than the second one (54%) (Fig 4). The high conservation of DBD II first Zinc finger sequences should be noted (Fig 4B). The distance between the first and second Zinc finger of the DBD I is greater in group β NRs. The three residues of the P-box reported to be responsible for specific contacts with the DNA response elements are identical in the analysed cestodes but differ between DBD I (EAK) and DBD II (EGG). The D-box of both domains seems to be specific to each group denoting different dimerisation preferences. LBD regions of the analysed cestodes NRs have little sequence conservation. The length of the putative hinge sequence and Ct regions are completely specific to each organism with variable sequence conservation. The LBD signature (Tτ) shows few amino acids with 100% conservation with a consensus sequence that departs from the consensus sequences reported. AF-2 regions are conserved in all sequences with a consensus of L (YF) (VSTL) (EQ) M, with a bias characteristic of each subfamily (Fig 4).

We also compared Eg2DBD sequences with EgHR3-like protein, the first *E*. *granulosus* nuclear receptor characterised [32]. EgHR3-like protein DBD domain shares high identity with the second DBD domain of Eg2DBDs differing in the number of residues of the second finger. The remaining sequence has low similarity.

### Phylogenetic studies

**Fig 5:**
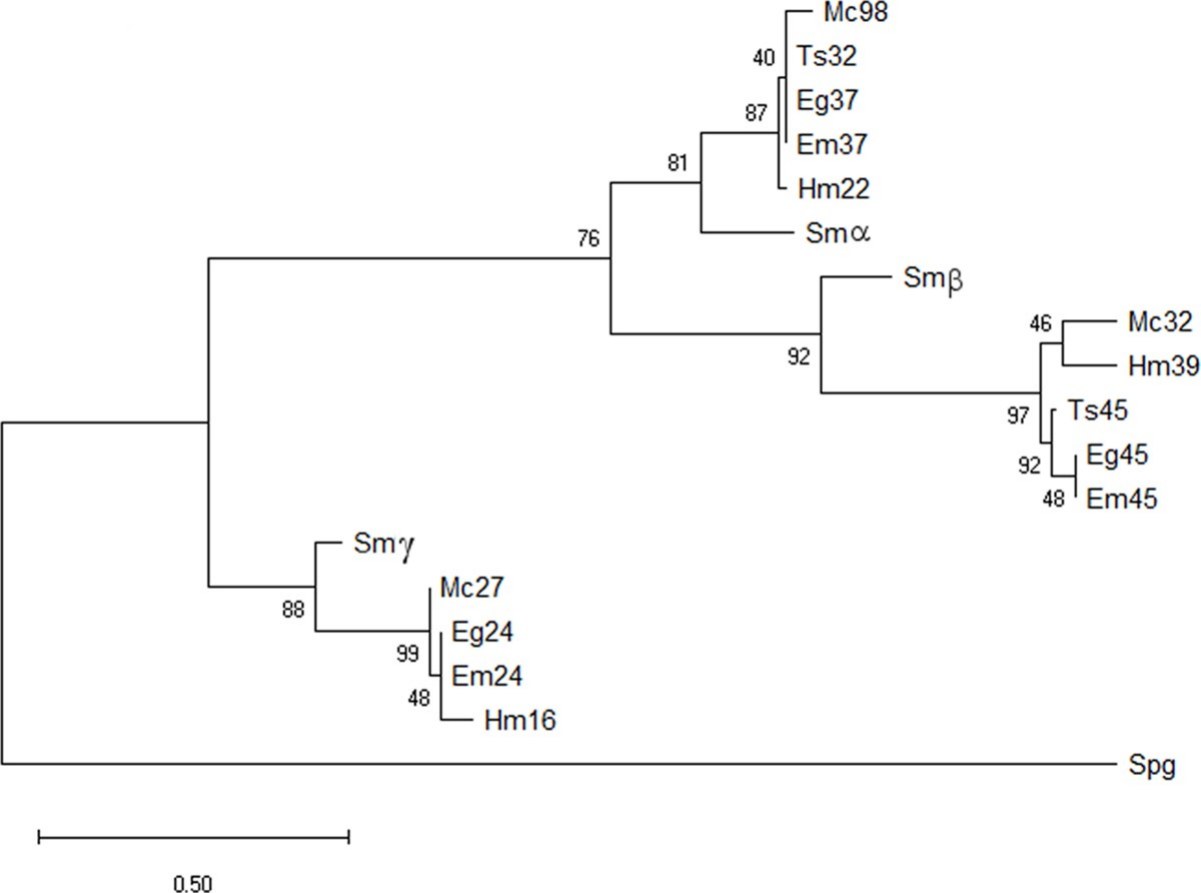
Maximum Likelihood phylogenetic tree from sequences of cestodes DNA binding domain I and II. Abbreviation and accession numbers of cestodes amino acid sequences are indicated in Fig 4. *S*. *mansoni* sequences abbreviations and Genbank accession numbers are: Smα: 2DBDα (AAR32910.1) Smβ: 2DBDβ (AAW88534.2); Smγ: 2DBDγ (AAW88550.2) Outgroup: *Amphimedon queenslandica* NR-2 (NP_001266221.1). Bootstrapping support values are indicated. Branch lengths are proportional to the genetic distances, as indicate by the scale bar representing 0.5 substitution per site.

The analysis performed by Wu and collaborators [25] indicated that 2DBD-NRs underwent two rounds of duplication in a common ancestor of the Platyhelminths giving rise to three genes. To support this conclusion, we performed a phylogenetic reconstruction employing representative cestode sequences and *S*. *mansoni* 2DBD nuclear receptors Sm2DBDα, Sm2DBDβ and Sm2DBDγ (Fig 5). DBD I and DBD II were employed to this purpose. The cestode species analysed express a set of 2DBD-NR proteins that broadly overlap with that of the related platyhelminth *S*. *mansoni* 2DBD NRs (Sm2DBDα, Sm2DBDβ and Sm2DBDγ) [9, 29].

## Discussion

Nuclear receptors belong to the transcription factor family that regulates development, homeostasis, differentiation, and reproduction in metazoans via control of gene expression. NRs in parasitic platyhelminths were first identified in *S*. *mansoni* [18]. Later, nuclear receptors were reported in *Taenia crassiceps*, *Opistorchis felineus* and mainly in *S*. *mansoni* [9, 38, 39–40]. Three nuclear receptors have been characterised in *Echinococcus* species, DAF-12-like, HR3-like, and estrogen-like receptors [32, 41–42]. The availability of *E*. *granulosus* sequences in the GeneDB database allowed us to search for PPARα-like nuclear receptor finding the members of the new 2DBD subfamily. The phylogenetic studies we carried out lead us to name the sequences annotated as Eg2DBDα (EgrG 000379600.1), Eg2DBDβ (EgrG 000458200) and Eg2DBDγ (EgrG 00240200). The GeneDB database refers to the product of these sequences as 2DBDγ nuclear receptor. In this sense, we suggest to change the actual nomination of the other cestodes 2DBD sequences analysed here.

In order to characterize these proteins we used the sequence data for cDNA amplification. Only one transcript could be amplified from the larval stage using specific primers. Although it is possible that there are errors in the annotated sequences of Eg2DBDβ and Eg2DBDγ that would hinder the hybridisation of the designed primers, other scenarios are also reasonable. On the one hand, considering that the synthesized cDNA represents the set of transcribed genes, it is possible that these receptors are not being transcribed in the larval stage of protoscoleces. On the other hand, it has been reported that in this larval stage, 33% of the genes are subject to alternative splicing, so it is also feasible that the genes corresponding to Eg2DBDβ and Eg2DBDγ undergo a process of this type in which case the primer hybridisation regions could be absent [43]. In this case, only Eg2DBDα isoform is expressed in protoscoleces. Finally, we cannot discard that low- level expression of these two receptors did not allow their amplification.

Likewise, the cloned fragment presented some differences with respect to the sequence annotated in the database. Since the DNA polymerase used in this stage is a high-fidelity enzyme for its proof-reading activity, we do not consider that these changes are attributable to an error of the enzyme, but to the presence of an isoform of Eg2DBDα. Among these differences, four of them represent an amino acid change, while at position 502 of the protein sequence, the absence of 22 amino acids was observed. The analysis of the gene structure of the annotated sequence showed that the deleted region is encoded by the 3' end of the fifth exon, representing a splice with the use of an alternative 5' site. Alternative splice events can be commonly classified into seven types: intron retention (IR), exon skipping (ES), alternative 3'-site (A3'), alternative 5'-site (A5'), first alternative exon (FAE), last alternative exon (LAE) and mutually exclusive exon (MEE) [44]. In turn, it has been observed that the general patterns of alternative splicing vary between species, tissues and stages of development [45–46]. For *E*. *granulosus* protoscolex larval stage, the proportion of the seven alternative splice events is as follows: IR 39%, A5' 21%, A3' 17%, ES 16%, FAE 4%, LAE 2% and MEE 1% [43]. Accordingly, the type of alternative splicing event (A5') evidenced at position 502 of the protein sequence is one of the most frequent at this stage of the development of the parasite under study. Interestingly, NCBI genome project reported a protein embryonic gonad from *E*. *granulosus* (XP_024349575.1) with 70% similarity with the cloned Eg2DBDα. This protein seems to be a second isoform expressed in the adult stage of the parasite.

Bioinformatics tools were employed to characterise the *Eg2DBD*α cloned region. Specific nuclear receptor motifs, as well as consensus signal sequences, were identified. The P-box motif is critical for DNA-binding specificity and is highly conserved within the family of nuclear receptors. The P-box, first labelled as "Proximal box", was identified by the Evans team as the essential amino acids for DNA binding [47]. Three residues of the P-box, the first, the second and the fifth residues, distinguish nuclear receptors and discriminate the central two nucleotides of the half recognition element of DNA. This triad was initially reported in P-boxes of Glucocorticoid receptor "GSV", Estrogen receptor "EGA", Thyroid hormone receptor "EGG"; Fushi Tarazu Factor-1 "ESG", verbA and knirps receptors "EGS" and Estrogen related receptors "EAA" [48–49].

Interestingly, the triad “EAK” from the first DBD of the Eg2DBD-NRs P-boxes (EACKK) seems to be specific to the 2DBD-NR subfamily. An exhaustive work with 57 P-box variants and various DNA repeat elements, which differ in sequences at the two central bases was reported [49]. According to their results, we propose that the target sequence for EAK P-box could be AGG(C/G)CA. On the other hand, the "EGG" P-box triad of the second DBDs from Eg2DBD-NRs is identical to most members of the NR subfamily I, for instance, Thyroid hormone receptor, PPARs and ROR. In general, receptors of this subfamily contain an EGG P-box sequence and recognise DNA elements with the nucleotide sequence of AGGTCA. Following the previous argument, the putative target sequence for the second DBD P-box from Eg2DBD-NRs could be AGG(T/C/A)CA.

A second consensus motif has been defined in the DNA-binding domain, called D-box, which is a 5-amino acid loop that defines a strong dimerisation interface for homodimer formation and contributes, to a much lesser extent, to heterodimer stabilisation [11,49]. Eg2DBDα D-boxes, "PSGGN" and "VGCKDN", differ from D-boxes reported by Umesono and Evans for GR and ER/TR subfamilies [47]. These regions are also specific for each cestode subtype indicating different dimerisation behaviour. *M*. *corti* 2DBD-NRs are an exception since Mc2DBDα and Mc2DBDβ D-boxes of the first DBD differ from those of the other members of the subfamily.

The presence of two DBDs in the same receptor is a novel finding. There are no precedents that allow us to generate a hypothesis about how two different P-boxes would act in the same protein. The fact that each DBD has specific P- and D-boxes could indicate that each domain acts independently. The 3D model of Eg2DBDα DNA binding domains bound to DNA suggests that only one DNA binding domain is able to specifically recognize a DNA response element. The other DBD could contribute to the stabilisation of the complex.

Based on phylogenetic analysis, it was suggested that 2DBD-NRs have a close functional or evolutionary relationship with NR subfamily I [29]. We consider that the known ligands of subfamily I members, fatty acids, prostaglandins, cholesterol and retinoic acid could be suitable ligands for *E*. *granulosus* 2DBD-NRs. Of particular interest are fatty acids, since the parasite does not synthesize them *de novo*. Consequently, these molecules should be obtained from the host and distributed among cellular organelles by fatty acid transporters [23,50]. Recently, our research group has demonstrated the presence of the fatty acid-binding protein, EgFABP1, in the nuclei of protoscoleces cells, suggesting that it could be involved in fatty acid transport to the nucleus and delivery to PPAR-like receptors [50]. The identified Eg2DBDα-NR could be a good candidate to interact with EgFABP1.

NRs characteristically possess a major NLS (termed NL1) that is composed of basic amino acids at the C-terminal region of the DNA-binding domain [36]. Nuclear localisation signals are an apparently diverse set of sequences with a generally polybasic characteristic. The best characterised NLS of proteins is exemplified by the “classical” NLS of the SV40-T antigen, which is identifiable in the primary sequence of a protein as a series of basic residues in the form K(K/R)X(K/R) [32,51–52]. However, several NLSs that do not completely fit this consensus have been identified. Functional NLS have been reported for glucocorticoid receptor (GR) as KKKIK, for mineralocorticoid receptor (MR) KKX2K, KRK for CRBP II, and KRR for FABP4 [34–37]. A second NLS (termed NL2) was also identified within GR and estrogen receptor LBDs [36]. A new class of NLSs termed NL0 (K/R X7RR) requiring a serine/threonine motif, was reported in MR [36]. Taking together these data, we propose two putative NLSs composed by residues ^326^-KRX2R-^331^ and ^419^-RRK-^422^ located at the hinge domain. The same location has been reported in other NRs [12,53].

SUMOylation has been reported to regulate protein subcellular localisation, protein–DNA binding, protein–protein interactions, transcription, DNA repair and genome organisation [54]. SUMO activating enzyme has been identified in *E*. *granulosus* (GenBank accession number CDS16643.1) suggesting that the SUMOylation pathway could be active. Two consensus SUMOylation sequences were identified in Eg2DBDα isoform at the hinge and C-terminal region. This post-translational modification has been reported for RORα, ERα and PPARα at the hinge region [55–56] and for PPARγ, LXRβ and PPARα at the LBD domain [57–59]. SUMOylation in the C-terminal AF-2 region is now viewed as a critical mechanism regulating the balance between trans-activating and trans-repressive functions of NRs [12]. In this sense, Eg2DBDα could undergo a similar regulatory mechanism.

The NRs have become attractive targets for the development of drugs of small molecular size, due to the lipophilic nature of their ligands and their ability to modulate the expression of multiple genes in the same pathway [60]. Parasitic helminth NRs have been proposed as a target for a new therapeutic strategy different from the known anthelmintic drugs, such as the benzimidazoles, whose target is tubulins [60]. In addition, it should be noted that approximately 13% of drugs approved by the FDA interact with NRs [61]. Taking into account that vertebrates lack 2DBD-NRs, these atypical proteins seem to be good putative targets as novel drugs.

Considering the previously mentioned, the elucidation of 2DBD-NRs targets in *E*. *granulosus* could allow for the discovery of new and specific pathways differing from those of their host.

## Conclusions

*Echinococcus granulosus* Eg2DBDα, Eg2DBDβ and Eg2DBDɣ proteins are members of the new 2DBD-NRs subfamily. These proteins possess the typical architecture and conserved motifs of NRs. Members of this subfamily are present in other cestodes. These proteins, as well as those analysed from cestodes are phylogenetically related to *S*. *ma*nsoni 2DBD-NRs subtypes α, β and ɣ supporting the evolutionary relationship with NR subfamily I suggested by Wu et al. [9].

Despite the fact that *E*. *granulosus* database reports three different Eg2DBDs, only one of them could be amplified under the conditions tested. The transcript obtained corresponds to an isoform of the GeneDB database gene Egr_000379600.1. It could be the most expressed 2DBD-NR in the protoscolex larval stage. The characterised Eg2DBDα isoform might be a good candidate to interact with EgFABP1. Furthermore, this isoform contains putative NLS and SUMOylation signals.

The high similarity among the DBD domains of the Eg2DBD-NRs does not allow elucidation of whether they could have different targets, however, they probably have specific activation mechanisms since the LBDs are poor conserved among them. This fact should give rise to specific dimerisation behavior, probably involving different ligands, activators and repressors. The obtained 3D model suggests a new hypothesis of how two different P-boxes would act on a target gene. Identification of putative ligands, dimerisation behaviour and target genes will be the focus of future work to further characterise the function of these particular receptors.

## References

[1] Jensen EV. “On the Mechanism of Estrogen Action.” Perspectives in Biology and Medicine. 1962;6: 47–60. 10.1353/pbm.1963.0005 13957617

[2] Evans RM, David J, Mangelsdorf DJ. Nuclear Receptors, RXR, and the Big Bang. Cell. 2014;157: 255–266. 10.1016/j.cell.2014.03.012 24679540 PMC4029515

[3] Weikum ER, Liu X, Ortlund EA. The nuclear receptor superfamily: A structural perspective. Protein Sci. 2018;27:1876–1892. 10.1002/pro.3496 30109749 PMC6201731

[4] Mangelsdorf DJ, Evans RM. The RXR heterodimers and orphan receptors. Cell. 1995;83: 841–850. 10.1016/0092-8674(95)90200-7 8521508

[5] Enmark E, Gustafsson JA. Orphan nuclear receptors: the first eight years. Mol Endocrinol. 1996;10: 1293–307. 10.1210/mend.10.11.8923456 8923456

[6] Bridgham JT, Eick GN, Larroux C, Deshpande K, Harms MJ, Gauthier ME, Ortlund EA, Degnan BM, Thornton JW. Protein evolution by molecular tinkering: diversification of the nuclear receptor superfamily from a ligand-dependent ancestor. PLoS Biol. 2010;8i: e1000497. 10.1371/journal.pbio.1000497 PMC2950128 20957188

[7] Escriva H, Bertrand S, Laudet V. The evolution of the nuclear receptor superfamily. Essay Biochem. 2004;40: 11–26. 10.1042/bse0400011 15242336

[8] Laudet V, Auwerx J, Gustafsson JA, Wahli W. A unified nomenclature system for the nuclear receptor superfamily. Cell. 1999;97: 161–163. 10.1016/s0092-8674(00)80726-6 10219237

[9] Wu W, Niles EG, El-Sayed N, Berriman M, LoVerde PT. *Schistosoma mansoni* (Platyhelminthes, Trematoda) nuclear receptors: sixteen new members and a novel subfamily. Gene. 2006;366: 303–15. 10.1016/j.gene.2005.09.013 16406405

[10] Giguère V, Hollenberg SM, Rosenfeld MG, Evans RM. Functional domains of the human glucocorticoid receptor. Cell. 1986;46: 645–52. 10.1016/0092-8674(86)90339-9 3742595

[11] Germain P, Staels B, Dacquet C, Spedding M, Laudet V. Overview of nomenclature of nuclear receptors. Pharmacol. Rev. 2006;58: 685–704. 10.1124/pr.58.4.2 17132848

[12] Pawlak M, Lefebvre P, Staels B. General molecular biology and architecture of nuclear receptors. Curr Top Med Chem. 2012;12:486–504. 10.2174/156802612799436641 22242852 PMC3637177

[13] Laudet G, Adelmant G. Nuclear receptors. Lonesome orphans. Curr Biol. 1995;5: 124–126. 10.1016/s0960-9822(95)00031-5 7743173

[14] Bain D, Heneghan AE, Connaghan-Jones KD, Miura MT. Nuclear receptor structure: implications for function. Annu Rev Physiol. 2007; 69: 201–220. 10.1146/annurev.physiol.69.031905.160308 17137423

[15] Melvin VS, Roemer SC, Churchill ME, Edwards DP. The C-terminal extension (CTE) of the nuclear hormone receptor DNA binding domain determines interactions and functional response to the HMGB-1/-2 co-regulatory proteins. J Biol Chem. 2002;277: 25115–25124. 10.1074/jbc.M110400200 12006575

[16] Wurtz JM, Bourguet W, Renaud JP, Vivat V., Chambon P., Moras D, et al A canonical structure for the ligand-binding domain of nuclear receptors. Nat Struct Biol. 1996;3: 87–94. 10.1038/nsb0196-87 8548460

[17] Thompson RCA. Biology and Systematics of *Echicococcus* In: *Echinococcus* and Hydatid Disease. CAB International Publication, Oxford University Press, Oxford, England 1995

[18] Escriva H, Safi R, Hanni C, Anglois MC, Saumitou-Aprade P. Stehelin, et al Ligand binding was acquired during evolution of nuclear receptors. Proc Natl Acad Sci USA. 1997;94: 6803–6808. 10.1073/pnas.94.13.6803 9192646 PMC21239

[19] Freebern WJ, Niles EG, LoVerde PT. RXR-2, a member of the retinoid x receptor family in *Schistosoma mansoni*. Gene, 1999; 233: 33–8. 10.1016/s0378-1119(99)00161-4 10375618

[20] Freebern WJ, Osman A, Niles EG, Christen L, LoVerde PT. Identification of a cDNA encoding a retinoid X receptor homologue from *Schistosoma mansoni*. Evidence for a role in female-specific gene expression. J Biol Chem. 1999;274: 4577–85. 10.1074/jbc.274.8.4577 9988692

[21] De Mendonça RL, Escriva H, Bouton D, Zelus D, Vanacker JM, Bonnelye E, Cornette J, Pierce RJ, Laudet V. Structural and functional divergence of a nuclear receptor of the RXR family from the trematode parasite Schistosoma mansoni. Eur J Biochem. 2000; 267: 3208–19. 10.1046/j.1432-1327.2000.01344.x 10824105

[22] De Mendonça RL, Bouton D, Bertin B, Escriva H, Noël C, Vanacker JM, Cornette J, Laudet V, Pierce RJ. A functionally conserved member of the FTZ-F1 nuclear receptor family from Schistosoma mansoni. Eur J Biochem. 2002; 269: 5700–11. 10.1046/j.1432-1033.2002.03287.x 12423370

[23] Esteves A, Dallagiovanna B, Ehrlich R. A developmentally regulated gene of *Echinococcus granulosus* codes for a 15.5-kilodalton polypeptide related to fatty acid binding proteins. Mol Biochem Parasitol. 1993;58: 215–222. 10.1016/0166-6851(93)90043-w 8479446

[24] Sievers F, Wilm A, Dineen D, Gibson TJ., Karplus K, Li W, et al Fast, scalable generation of high-quality protein multiple sequence alignments using Clustal Omega. Mol Syst Biol 2011;7: 539 10.1038/msb.2011.75 21988835 PMC3261699

[25] Milburn D, Laskowski RA, Thornton JM. Sequences annotated by structure: a tool to facilitate the use of structural information in sequence analysis. Prot. Eng. 1998;11: 855–859. 10.1093/protein/11.10.855 9862203

[26] Roy A, Xu D, Poisson J, Zhang Y. A Protocol for Computer-Based Protein Structure and Function Prediction. J Vis Exp. 2011;57: e3259. 10.3791/3259 PMC3308591 22082966

[27] Kumar S., Stecher G., Li M., Knyaz C., and Tamura K. MEGA X: Molecular Evolutionary Genetics Analysis across computing platforms. Molecular Biology and Evolution. 2018;35:1547–1549. 10.1093/molbev/msy096 29722887 PMC5967553

[28] Jones D.T., Taylor W.R., and Thornton J.M. The rapid generation of mutation data matrices from protein sequences. Computer Applications in the Biosciences. 1992;8: 275–282. 10.1093/bioinformatics/8.3.275 1633570

[29] Wu W, Niles EG, Hirohisa Hirai H, LoVerde PT. Evolution of a novel subfamily of nuclear receptors with members that each contain two DNA binding domains. BMC Evolutionary Biology. 2007; 7: 27 10.1186/1471-2148-7-27 17319953 PMC1810520

[30] Laudet V, Hanni C, Coll J, Catzeflis F, Stehelin D. Evolution of the nuclear receptor gene superfamily. EMBO J. 1992;1: 1003–1013. 10.1002/j.1460-2075.1992.tb05139.x PMC556541 1312460

[31] Brelivet Y, Kammerer S, Rochel N, Poch O, Moras D. Signature of the oligomeric behaviour of nuclear receptors at the sequence and structural level. EMBO Rep. 2004;5: 423–9. 10.1038/sj.embor.7400119 15105832 PMC1299030

[32] Yang M, Li J, Wu J Wang H, Guo B, Wu C, et al Cloning and characterization of an Echinococcus granulosus ecdysteroid hormone nuclear receptor HR3-like gene. Parasite 2017;24: 36 10.1051/parasite/2017037 28971798 PMC5625357

[33] Dingwall C, Laskey RA. Nuclear targeting sequences a consensus? Trends Biochem. Sci. 1991;16: 478–481. 10.1016/0968-0004(91)90184-w 1664152

[34] Picard D, Yamamoto KR. Two signals mediate hormone-dependent nuclear localization of the glucocorticoid receptor. EMBO J. 1987;6: 3333–3340. 3123217 10.1002/j.1460-2075.1987.tb02654.x PMC553788

[35] Sessler RJ, Noy N. A ligand-activated nuclear localization signal in cellular retinoic acid binding protein-II. Mol Cell. 2005;18: 343–53. 10.1016/j.molcel.2005.03.026 15866176

[36] Walther RF, Atlas E, Carrigan A, Rouleau Y, Edgecombe A, Visentin L, et al A Serine/Threonine-rich motif is one of three nuclear localization signals that determine unidirectional transport of the mineralocorticoid receptor to the nucleus. J Biol Chem. 2005;280: 17549–17561. 10.1074/jbc.M501548200 15737989

[37] Ayers SD, Nedrow KL. Gillilan RE, Noy N. Continuous nucleocytoplasmic shuttling underlies transcriptional activation of PPARγ by FABP4. Biochemistry. 2007;46: 6744–6752. 10.1021/bi700047a 17516629

[38] Escobedo G, Larralde C, Chavarria A, Cerbón MA, Morales-Montor J. Molecular mechanisms involved in the differential effects of sex steroids on the reproduction and infectivity of *Taenia crassiceps*. J Parasitol. 2004;90: 1235–1244. 10.1645/GE-297R 15715212

[39] Pakharukova MY, Ershov NI, Vorontsova EV, Shilov AG., Merkulova TI, Mordvinov VA. Identification of thyroid hormone receptor homologs in the fluke Opisthorchis felineus (Platyhelminthes). Mol Biochem Parasitol. 2014;194: 64–68. 10.1016/j.molbiopara.2014.04.009 24798031

[40] Wu W, LoVerde PT. Nuclear hormone receptors in parasitic helminths. Mol Cell Endocrinol. 2011;334: 56–66. 10.1016/j.mce.2010.06.011 20600585 PMC2974807

[41] Förster S, Günthel D, Kiss F, Brehm K. Molecular characterisation of a serum-responsive, DAF-12-like nuclear hormone receptor of the fox-tapeworm Echinococcus multilocularis. J Cell Biochem. 2011; 112: 1630–1642. 10.1002/jcb.23073 21328613

[42] Nicolao MC, Elissondo MC, Denegri GM, Goya AB, Cumino AC. *In vitro* and *in vivo* effects of tamoxifen against larval stage Echinococcus granulosus. Antimicrob Agents Chemother. 2014;58: 5146–5154. 10.1128/AAC.02113-13 24936598 PMC4135819

[43] Liu S, Zhou X, Hao L, Piao X, Hou N, Chen Q. Genome-wide Transcriptome analysis reveals extensive alternative splicing events in the protoscoleces of Echinococcus granulosus and Echinococcus multilocularis. Front Microbiol. 2017;8: 929 10.3389/fmicb.2017.00929 28588571 PMC5440512

[44] Black DL. Mechanisms of alternative pre-messenger RNA splicing. Ann Rev Biochem. 2003;72: 291–336. 10.1146/annurev.biochem.72.121801.161720 12626338

[45] Wang ET, Sandberg R, Luo S, Khrebtukova I, Zhang L, Mayr C, Burge CB. Alternative isoform regulation in human tissue transcriptomes. Nature, 2008;456: 470–476. 10.1038/nature07509 18978772 PMC2593745

[46] Gibilisco L, Zhou Q, Mahajan S, Bachtrog D. Alternative splicing within and between Drosophila species, sexes, tissues, and developmental stages. PLoS Genetics. 2016;12, e1006464 10.1371/journal.pgen.1006464 27935948 PMC5147784

[47] Umesono K, Evans RM. Determinants of target gene specificity for steroid/thyroid hormone receptors. Cell. 1989;57: 1139–1146. 10.1016/0092-8674(89)90051-2 2500251

[48] Luisi BF, Xu WX, Otwinowski Z, Freedman LP, Yamamoto KR, Sigler PB. Crystallographic analysis of the interaction of the glucocorticoid receptor with DNA. Nature. 1991;352: 497–505. 10.1038/352497a0 1865905

[49] Zechel C, Shen Xi-Q, Chambon P, Hinrich Gronemeyer H. Dimerization interfaces formed between the DNA binding domains determine the cooperative binding of RXR/RAR and RXR/TR heterodimers to DR5 and DR4 elements. EMBO J. 1994;13: 1414–1424. 8137825 10.1002/j.1460-2075.1994.tb06395.x PMC394959

[50] Alvite G, Esteves A. *Echinococcus granulosus* fatty acid binding proteins subcelullar localization. Exp Parasitol. 2016;164: 1–4 10.1016/j.exppara.2016.02.002 26873273

[51] Kalderon D, Roberts BL, Richardson WD, Smith AE. A short amino acid sequence able to specify nuclear location. Cell. 1984;39: 499–509. 10.1016/0092-8674(84)90457-4 6096007

[52] Hodel MR, Corbett AH, Hodel AE. Dissection of a nuclear localization signal. J Biol Chem. 2001;276: 1317–1325. 10.1074/jbc.M008522200 11038364

[53] Robbins J, Dilworth SM, Laskey RA, Dingwall C. Two interdependent basic domains in nucleoplasmin targeting sequence: identification of a class of bipartite nuclear targeting sequence. Cell. 1991;64: 615–623. 10.1016/0092-8674(91)90245-t 1991323

[54] Hickey CM, Wilson NR, Hochstrasser M. Function and regulation of SUMOproteases. Nat Rev Mol Cell Biol. 2012;13: 755–766. 10.1038/nrm3478 23175280 PMC3668692

[55] Sentis S, Le Romancer, M, Bianchin C, Rostan M-C, Corbo L. Sumoylation of the estrogen receptor hinge region regulates its transcriptional activity. Mol. Endocrinol. 2006;19: 2671–2684. 10.1210/me.2005-0042 15961505

[56] Pourcet B, Pineda-Torra I, Derudas B, Staelsm B, Glineurm, C. SUMOylation of human peroxisome proliferator-activated receptor alpha inhibits its trans-activity through the recruitment of the nuclear corepressor NCoR. J Biol Chem. 2010;285: 5983–5992. 10.1074/jbc.M109.078311 19955185 PMC2825392

[57] Pascual G, Fong AL, Ogawa S, Gamliel A, Li AC, Perissi V, et al A SUMOylation-dependent pathway mediates transrepression of inflammatory response genes by PPAR-gamma. Nature. 2005;437: 759–763. 10.1038/nature03988 16127449 PMC1464798

[58] Venteclef N, Jakobsson T, Ehrlund A, Damdimopoulos A, Mikkonen L, Ellis E, et al GPS2-dependent corepressor/SUMO pathways govern anti-inflammatory actions of LRH-1 and LXRbeta in the hepatic acute phase response. Genes Dev. 2010;24: 381–395. 10.1101/gad.545110 20159957 PMC2816737

[59] Treuter E, Venteclef N. Transcriptional control of metabolic and inflammatory pathways by nuclear receptor SUMOylation. Biochim Biophys Acta. 2011;1812: 909–918. 10.1016/j.bbadis.2010.12.008 21172431

[60] Wang Z, Schaffer NE, Kliewer SA, Mangelsdorf DJ. Nuclear receptors: emerging drug targets for parasitic diseases. J. Clin. Invest. 2017;127: 1165–1171. 10.1172/JCI88890 28165341 PMC5373876

[61] Overington JP, Al-Lazikani B, Hopkins AL. How many drug targets are there? Nat. Rev Drug Discov. 2006;5: 993–996. 10.1038/nrd2199 17139284

